# The choice of anaesthesia for glioblastoma surgery does not impact the time to recurrence

**DOI:** 10.1038/s41598-020-62087-8

**Published:** 2020-03-27

**Authors:** Stefan J. Grau, Mario Löhr, Valeria Taurisano, Herbert Trautner, Marco Timmer, Stephanie G. Schwab, Jürgen Hampl, Thorsten Annecke

**Affiliations:** 1 University of Cologne, Medical Faculty and University Hospital of Cologne, Center for Neurosurgery, Dept of General Neurosurgery, Kerpener Strasse 62, D-50937 Cologne, Germany; 2 University of Cologne, Medical Faculty and University Hospital of Cologne, Department of Anaesthesiology and Intensive Care Medicine, Kerpener Strasse 62, D-50937 Cologne, Germany; 3 University Hospital of Wuerzburg, Department of Neurosurgery, Josef Schneider Strasse 11, D-97080 Wuerzburg, Germany; 4 University Hospital of Wuerzburg, Department of Anaesthesiology, Oberdürrbacher Strasse 6, D- 97080 Wuerzburg, Germany

**Keywords:** CNS cancer, CNS cancer

## Abstract

Anaesthetics used during cancer surgery may influence tumour cells and immunological response. The aim of this study was to evaluate a potential influence of the anaesthetic method (inhaled anaesthetics versus total-intravenous anaesthesia using propofol) on recurrence-free and overall survival in glioblastoma patients. We retrospectively identified patients undergoing resection of contrast enhancing glioblastoma under general anaesthesia followed by standard adjuvant treatment between January 2010 and February 2017 at two University Hospitals. Matched pairs of patients receiving either balanced with volatile anaesthetics or total intravenous anaesthesia were generated according to the known prognostic factors (extent of resection, methyl-guanine-methyl-transferase (MGMT) promoter methylation, age, Karnofsky performance score). Groups were compared using chi-square and Whitney-Man-U test. Time to recurrence was calculated using Kaplan Meier estimates. Log Rank test was used to assess the influence of the anaesthetic method. One hundred and fifty-eight (79:79) patients were included. Groups showed no significant difference in recurrence-free (volatiles: 8.0 (95% CI 6.5–9.8) vs. propofol: 8.4 (95% CI 7.9–10.1) months; p = 0.54) or overall survival (propofol: 17.4 (95% CI 14.0–20.7) vs. volatiles: 16.9 (95% CI 13.9–20.1) months; p = 0.85). In contrast to potential beneficial effects in some other solid tumours, the choice of anaesthetic method had no impact on survival in patients with glioblastoma in a well-defined cohort.

## Introduction

In glioblastomas WHO IV (GBM) complete resection of the contrast-enhancing tissue positively influences progression-free and overall survival. Standard adjuvant treatment comprises radio-chemotherapy followed by temozolomide chemotherapy.

Surgical stress with subsequent neuroendocrine and inflammatory reactions may suppress cell-mediated immune responses and thus render the perioperative period a vulnerable time, which can be significantly modulated by different anaesthetic techniques^1,2^. In GBM, also the interaction of microglia, macrophages and the tumor itself may be affected. Therefore, there is growing interest in potential effects of anaesthesia on tumour progression and outcome in cancer patients^3,4^. While numerous studies have focused on surgical techniques and the extent of glioblastoma resection, the influence of the medication used for anaesthesia during surgery on patient outcome remains largely unclear, although the substances used (e.g. propofol, fluranes, opioids, benzodiazepines) are capable of passing the blood-brain barrier and have pleiotropic effects.

Differential effects of various anaesthetic agents on tumour cells have been demonstrated *in vitro*^5–7^. Regarding glioma cells, propofol could induce apoptosis and suppresses proliferation and invasion. In contrast, increased proliferation and decreased apoptosis was shown for isoflurane in human glioblastoma stem cells. However, experiments have not been consistent, depending on different models and cell lines used^8^. At a clinical level, initial studies showed that the anaesthetics used may have significant impact on the patient’s further course in solid tumours, suggesting some benefit with regional anaesthesia and local anaesthesia avoiding opioids and volatile anaesthetics. Indeed the hypnotic agent propofol has shown anticancer effects *in vitro* and *in vivo*^9,10^.

All substances used for brain tumour surgery may penetrate the blood-brain barrier and may thus exhibit effects on the tumour microenvironment. Since an effect on glioma cells and the patients´ further course may therefore be supposed, and if present, would be highly relevant in a rapidly progressive and fatal disease, we investigated the influence of the chosen anaesthetics on the prognosis of patients with GBM.

The effect of a total intravenous anaesthesia with propofol, compared to a balanced anaesthesia with volatile anaesthetics, both in conjunction with opioid supplementation, has not been investigated prospectively in patients with GBM. Since propofol has shown anticancer effects in different investigations, our hypothesis was that it would be superior to a balanced technique using volatile anaesthetics.

## Methods

### Study design

This is a retrospective cohort study, performed to analyse a potential impact of the anaesthetic method on the patients’ outcome with regard to recurrence-free survival. This study adheres to the applicable CONSORT guidelines (STROBE) in compliance with the Declaration of Helsinki and was approved by the local ethical committee (Ethikkommission der Medizinischen Fakultaet, University of Cologne, approval no. 16-417). No patient consent to review their medical records was required by the committee according to state law (§6 Abs.1 GDSG NRW).

### Patient selection

Patients undergoing resection of supratentorial primary glioblastoma (GBM, IDH wild type) between January 2010 and February 2017 were identified from clinical databases at two German University Hospitals. The following parameters were retrieved: age, gender, Karnofsky performance score (KPS), operation time and duration of anesthesia, type of anesthetics, dosage of intraoperative applied opioids, extent of resection (EOR) (total, almost total, subtotal), evaluated on post-op magnetic resonance imaging (MRI) +/− CE within 48 hours after surgery, methyl-guanin-methyl-transferase (MGMT) promoter methylation status, isocitrate-dehydrogenase (IDH) -1-mutation, mode of adjuvant treatment (radio-chemotherapy, radiotherapy, chemotherapy), medication for anaesthesia maintenance, time to progression (assessed by MRI according to RANO criteria), treatment after recurrence, and time of death.

Patients requiring intraoperative neurophysiological monitoring or awake craniotomy were excluded as were patients requiring emergency tumor-debulking.

Patients with IDH1 mutations were excluded due to their different characteristics as secondary glioblastomas resulting in a different prognosis. Furthermore, patients not receiving postoperative radio-chemotherapy either due to clinical trial participation or poor clinical status (KPS < 70) were not included in the analysis.

**Figure 1:**
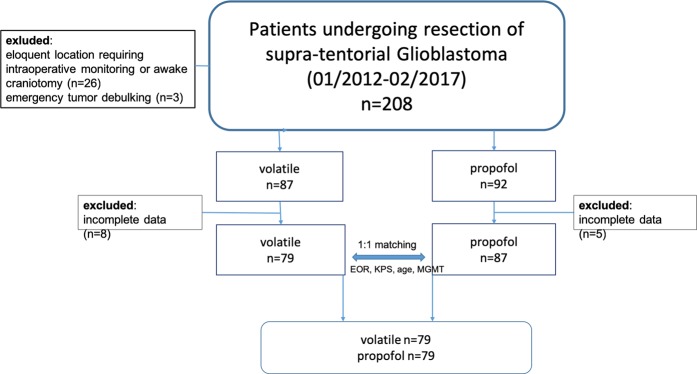
Flow-chart demonstrating patient selection and matching.

After identifying patients receiving volatile anaesthetics we formed a control group of patients receiving total intravenous anaesthesia by one-to-one exact matching based on well-known prognostic factors age, MGMT promoter methylation and extent of resection (EOR) (flow diagram, Fig. 1) in a 1:1 manner in order to generate an equal distribution of these known prognostic factors.

### Choice of anaesthesia

The choice of the anaesthetic agent depended solely on the experience and preference of the attending anaesthesiologists. After induction of general anaesthesia with propofol or thiopental, anaesthesia was maintained either solely by continuous infusion of propofol (*propofol group*), or by an inhalational anaesthesia with isoflurane, desflurane or sevoflurane (*volatile group*). Opioids (fentanyl, sufentanil or remifentanil) were added in each group. Despite the use of propofol or a volatile anaesthetic as a hypnotic component, anaesthesia treatment modalities, co-medication, hemodynamic stabilization and monitoring did not differ in either group.

Apart from short periods for induction and extubation (100% oxygen), standard operation procedures provided use of an inspiratory oxygen concentration of 30–40% in our circuit for both groups during the study period.

All patients received perioperative steroid treatment with dexamethasone.

### Clinical follow-up and definition of tumor progression

All patients underwent regular follow-up at outpatient clinics with a cMRI every three months. Progression was determined according to RANO criteria^11^.

### Statistical evaluation

Homogeneous distribution of the parameters was tested using Levene’s test. Between the groups (*volatile* vs. *propofol*) continuous variables were compared using Students’t-test for normally distributed variables, otherwise the Whitney-Mann-U test was used. Categorical variables were tested by chi-square statistics. Survival curves were estimated using the Kaplan Meier method. Influence of parameters (anaesthetic method, EOR, MGMT promoter methylation, KPS) was assessed using Log Rank test. Statistically significant factors were included in a Cox regression analysis. A p-value < 0.05 was considered statistically significant.

Glioblastomas are highly malignant brain tumours that cannot be cured to date. A major intention in treating these patients is to prolong the time until the tumour starts to grow again. This time span is called recurrence- or progression-free survival. Surgical removal of the visible tumour parts is one mainstay of multimodal glioblastoma therapy. During surgery various substances are applied for anaesthesia. These substances probably have an impact on tumour cell behaviour at least in a laboratory setting. The aim of this study was to evaluate whether these anaesthetic substances influence the time until tumour recurrence. Two balanced groups (receiving two different anaesthetic regimens) were compared with regard to recurrence-free and overall survival. The choice of the anaesthetic method (propofol vs. inhaled) did not influence time to recurrence or overall survival. These findings suggest that either anaesthetics have no impact on glioblastoma or the biological behaviour of these tumours is too aggressive to respond to perioperative influences.

## Results

### Treatment parameters and descriptive data

One hundred and fifty-eight patients undergoing complete resection of contrast-enhancing disease in glioblastoma under general anaesthesia were included: 79 patients receiving volatile maintained anaesthesia and 79 receiving total intravenous anaesthesia (Fig. 1).

Between the groups there were no significant differences in age (p = 0.732), Karnofsky performance score (p = 0.98), MGMT methylation status (p = 0.563) and gender distribution (p = 0.9). All tumors were IDH1-wild type.

Surgery was performed under 5-ALA fluorescence guidance. Gross total resection of contrast enhancing tumour tissue was proven in early post-op MRI with each group showing identical distribution (total resection n = 58, nearly total n = 20, subtotal n = 1).

The mean overall operation time was 193 (87–311) minutes and did not significantly differ between the groups (volatile 203 (118–311) vs. propofol 184 (87–272); p = 0.356). The mean overall duration of anesthesia was 282 (205–405) minutes and was also similar in both groups (volatile 293 (205–405) minutes vs Propofol 272 (210–369) minutes; p = 0.383) (Table 1). The type of intraoperative opioids varied between individual patients depending on the discretion of the treating anesthetist. However, dosage of the different opioids was comparable between groups (Table 1).

**Table 1:** Baseline characteristics of included patients.

Parameter	All	Volatiles	Propofol	p-value
Age median (range)	61 (28–86)	62 (28–86)	60 (33–80)	0.732
KPS median (range)	90 (70–100)	90 (70–100)	90 (80–100)	0.98
Mean operative time (min; range)	193 (87–311)	203 (118–311)	184 (87–272)	0.356
duration of anesthesia (min; range)	282 (205–405)	293 (205–405)	272 (210–369)	0.383
Opioid doses for induction (mean; range)				
Sufentanil (µg)	35 (15–50)	35 (20–50)	35 (15–50)	0.79
Fentanyl (mg)	0.23 (0.2–0.5)	0.25 (0.2–0.5)	0.2 (0.2–0.025)	0.44
Opioid doses for maintenance (mean; range)				
Sufentanil (µg)	247 (100–400)	230 (100–280)	255 (100–400)	0.3
Remifentanil (µg)	2610 (400–7300)	2730 (400–7300)	2100 (1500–2100)	0.79
Piritramide (mg)	5 (3–10)	6 (3–10)	4 (3–5)	0.24
EOR (n)				N/A
Gross total resection	116	58	58	
Nearly total resection	40	20	20	
Subtotal resection	2	1	1	
MGMT prom. meth. n (%)	80/158 (50.6)	40/79 (50.6)	40/79 (50.6)	0.563
IDH1-mutation (n)	0	0	0	N/A
TMZ cycles (n; range)		4.2 (0–19)	4.4 (0–14)	0.52

Surgical complications occurred in eight (5%) patients comprising wound healing disorders in three, hydrocephalus requiring ventriculo-peritoneal shunt in one, postoperative ischemia (n = 2) and new postoperative seizures (n = 2).

Patients received a regular medication scheme including dexamethasone, with the latter being tapered until discharge.

All patients underwent standard radio-chemotherapy receiving 60 Gy with concomitant temozolomide at 75 mg/m^2^ followed by adjuvant temozolomide if MRI showed no progression six weeks after radiotherapy according to current guidelines. The number of treatment cycles did not differ (p = 0.320) between the groups. Characteristics are shown in Table 1.

Treatment after recurrence was re-resection followed by re-radiation and/or chemotherapy, re-irradiation, or chemotherapy only, or palliative care only. There were no statistically significant differences between the groups.

### Oncological outcome

**Figure 2:**
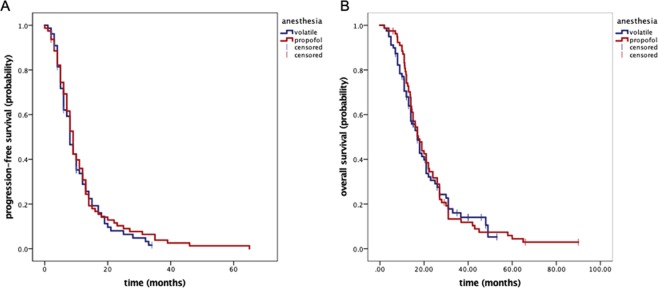
Kaplan-Meier plot documenting recurrence-free (**A**) and overall survival (**B**). There was no statistically significant difference between the groups (**A** p = 0.54; **B** p = 0.85).

The median follow-up period was 20 months (range 6–80). Median time to recurrence was 8.1 (95% CI 7.1–8.9) months for the entire cohort with 8.0 (95% CI 6.5–9.8) months in the volatiles and 8.4 (95% CI 7.9–10.1) months in the propofol group (p = 0.54; Fig. 2). Median overall survival was 17.1 (95% CI 14.7–19.3) months for the whole population with 17.4 (95% CI 14.0–20.7) months for the propofol and 16.9 (95% CI 13.9–20.1) months for the volatiles group (p = 0.85; Fig. 2).

MGMT promoter methylation was a significant prognostic factor for OS and PFS in both the entire cohort (OS p = 0.043; PFS p = 0.041) and the anaesthetic subgroups (volatiles OS p = 0.049; PFS 0.031; propofol OS p = 0.041, PFS p = 0.029) in univariate analysis. In MGMT subgroups (methylated and unmethylated) PFS and OS was also not altered by the choice of anaesthesia (data not shown).

In multivariate regression analysis only MGMT promotor methylation status (HR 1.4 95%CI 1.1–2.0; p = 0.019), gross total resection (HR 1.3 95%CI 1.1–1.9; p = 0.032) and age <70 (HR1.2 95%CI 1.1–1.8; p = 0.044) remained prognostic for progression-free survival.

## Discussion

Standard treatment for glioblastoma multiforme comprises maximal safe surgical resection of contrast enhancing tumour tissue followed by chemo-radiotherapy. Despite aggressive therapies the prognosis for these patients remains poor and is mainly influenced by MGMT promotor methylation and the extent of resection.

Despite the proven role of surgery in the treatment algorithm of many solid tumours, the influence of the anaesthetic intervention applied to perform surgery is usually neglected.

Several studies started to raise the question of whether the anaesthetic technique employed may indeed have a profound impact on tumour biology, metastasis formation, and thus the patients’ further course^10,12^.

Different anaesthetics are known to have pleiotropic immunological effects^13^. Volatile anaesthetics may protect against hypoxia and ischemia/reperfusion injuries in different organ systems^13,14^. Upregulation of hypoxia-inducible factor 1a (HIF-1a) seems to be involved in this context^14^. A recent *in vitro* study demonstrated that isoflurane upregulated HIF-1a in a prostate cancer cell line, which could not be demonstrated with propofol^5^.

At a clinical level, a recent study showed that the choice of anaesthesia significantly influenced the patients’ further prognosis in surgically treated solid tumors^10^. The underlying reasons for this impact remain to be elucidated in detail. However, initial data show that anaesthetics can profoundly influence tumour cell biology itself as well as the patient’s immune system, thus altering the patient’s immune response to tumour cells^1,2,9^.

In the present study, we chose a narrowly defined cohort of patients with GBM to measure a potential impact of volatiles on the patients’ further course. By matching the cohorts for the well-known prognostic factors EOR, MGMT promoter methylation, age and KPS, we aimed to investigate the anaesthetic propofol versus a volatile anaesthetic agent as the single differing factor. Due to logistic institutional factors and the different availability of volatile anesthetics, isoflurane, sevoflurane and desflurane had to be summarized as “inhalational group” despite some substance specific difference on tumor biology and immune function may exist^8^. However, a recent retrospective study showed no difference in overall or progression free survival if desflurane or isoflurane have been used for glioblastoma surgery^15^. As a limitation, due to the retrospective nature of the study, variables have not been strictly controlled, but standard operation procedures have been availiable during the study period, making course of action comparable between both groups.

Nitrous oxide was not used in either group during the study period. Also, anesthesia duration was not different in both groups. As a possible limitation of our study, different types of opioids were used intraoperatively in individual patients in both groups. However, the mean dosage of the specific opioids used was not different between groups. Due to the retrospective nature, we were not able to evaluate the use of opioids after hospital discharge in both groups.

Our entire study population showed progression-free and overall survival rates in close accordance with studies involving larger numbers, indicating a very representative cohort^15^. Given the fact that the impact of these prognostic factors remained unchanged in both subgroups, this underlines the good balance of the cohort.

No tendency towards a positive or negative influence of volatiles compared to intravenous agents in progression-free survival was observed, since curves showed an identical shape and course. One might criticize the cohort size as being too small to reveal smaller effects; however, the completely identical course until progression, even when potentially calculated for larger cohorts, would make a clinically relevant effect highly improbable. Our observation is in line with a recent retrospective analysis comparing high-grade glioma patients receiving propofol or sevoflurane for maintainance which also showed no significant difference in progression free or overall survival^16^. However, a standardized therapy for glioblastomas is only defined for primary treatment. The value of any second-line therapy after tumor relapse is controversially discussed and its application varies strongly between different countries and centers. Therefore data regarding overall survival are subject to a certain bias.

These findings may either document the absence of a relevant effect of the anaesthetic technique, or may be mainly explained by the aggressiveness of glioblastoma, resulting in a poor prognosis despite any potential influence of anaesthetics. Furthermore, the volatile group comprised different substances, thus potentially disguising particular effects of single agents.

Taking a critical position, one may ask how any kind of drug, administered for only a few hours could substantially influence the behaviour of a tumour at all, which is subsequently treated by high-dose radiotherapy (lasting six weeks) and chemotherapy (lasting up to six months). From this point of view any relevant effect of anaesthetics on glioblastomas appears highly improbable, all the more since differences in survival reported in the study mentioned above^10^ delineated years after primary treatment in metastatic cancers. In glioblastomas survival rates exceeding two years and metastatic spread are extremely rare. Therefore, any potential modulation of these oncological aspects in glioblastomas may not emerge due to the fatal disease course and absence of systemic metastases in glioblastomas.

Besides its retrospective nature and its subsequent limitations, a justified point of discussion in this study may be the predominance of totally or nearly totally resected tumours. Pursuing the idea of a relevant differential effect of anaesthetics on tumour cells themselves, there may be little target tissue left in these patients. Then again, tumour boundaries in glioblastomas lie beyond the contrast-enhancing mass, with numerous tumour cells remaining in surrounding brain tissue, causing relapse.

## Conclusion

We show that the choice of the anaesthetic hypnotic agent for glioblastoma surgery does not influence the further course of patients undergoing standard therapy.

## Data Availability

The datasets generated during and/or analysed during the current study are not publicly available due to ethical requirements but are available from the corresponding author on reasonable request.
